# DNA nanostructure-directed assembly of metal nanoparticle superlattices

**DOI:** 10.1007/s11051-018-4225-3

**Published:** 2018-04-27

**Authors:** Sofia Julin, Sami Nummelin, Mauri A. Kostiainen, Veikko Linko

**Affiliations:** 1 Biohybrid Materials, Department of Bioproducts and Biosystems, Aalto University, Espoo, Finland; 2 HYBER Center of Excellence, Department of Applied Physics, Aalto University, Espoo, Finland

**Keywords:** Nucleic acids, DNA origami, Self-assembly, Metal nanoparticles, Plasmonics, DNA nanotechnology

## Abstract

Structural DNA nanotechnology provides unique, well-controlled, versatile, and highly addressable motifs and templates for assembling materials at the nanoscale. These methods to build from the bottom-up using DNA as a construction material are based on programmable and fully predictable Watson-Crick base pairing. Researchers have adopted these techniques to an increasing extent for creating numerous DNA nanostructures for a variety of uses ranging from nanoelectronics to drug-delivery applications. Recently, an increasing effort has been put into attaching nanoparticles (the size range of 1–20 nm) to the accurate DNA motifs and into creating metallic nanostructures (typically 20–100 nm) using designer DNA nanoshapes as molds or stencils. By combining nanoparticles with the superior addressability of DNA-based scaffolds, it is possible to form well-ordered materials with intriguing and completely new optical, plasmonic, electronic, and magnetic properties. This focused review discusses the DNA structure-directed nanoparticle assemblies covering the wide range of different one-, two-, and three-dimensional systems.

## Introduction

The advances in the field of structural DNA nanotechnology (Linko and Dietz 2013; Jones et al. 2015; Seeman and Sleiman 2017; Hong et al. 2017; Nummelin et al. 2018) and in particular the development of the DNA origami method (Rothemund 2006) have given rise to an extensive collection of structurally versatile microscale and nanoscale DNA structures. Based on their modularity and addressability, these rationally designed nanoarchitectures can be used in, e.g., nanoelectronics (Liu et al. 2011; Shen et al. 2015a), plasmonics (Kuzyk et al. 2012, 2014, 2016; Shen et al. 2018), optical devices (Gopinath et al. 2016; Pilo-Pais et al. 2017), molecular-scale precision measurements (Castro et al. 2017), drug-delivery applications (Douglas et al. 2012; Li et al. 2013; Perrault and Shih 2014; Linko et al. 2015; Surana et al. 2015; Ora et al. 2016; Auvinen et al. 2017), controlling chemical reactions (Linko et al. 2016; Grossi et al. 2017; Gothelf 2017), and super-resolution imaging (Graugnard et al. 2017). Beside these intriguing applications, DNA structures that are customizable in size and shape can also find uses in directing the self-assembly of nanoparticles (NPs) into highly ordered structures and superlattices as discussed in this review. Spatially well-ordered metal nanoparticles have unique electronic, magnetic, and optical properties, and hence, there is ever-increasing interest towards these kinds of nanomaterials (Ofir et al. 2008; Nie et al. 2010). In general, the construction of materials with nanometer-scale precision is rather challenging, but owing to the predictable and programmable DNA hybridization (i.e., Watson-Crick base pairing), the precise arrangement of molecular components at the nanoscale becomes feasible (Tan et al. 2011; Jones et al. 2015). The techniques for DNA-directed self-assembly of nanoparticles have therefore traditionally relied on the superior molecular recognition properties of the DNA, but there are also strategies taking advantage of electrostatic or other non-specific interactions.

DNA-based self-assembly of nanoparticles was pioneered by Mirkin, Alivisatos, and co-workers in 1996 (Mirkin et al. 1996; Alivisatos et al. 1996), and during the last two decades, a wide spectrum of arrangements of nanoparticles have been achieved using DNA molecules with complementary sequences as linkers (Macfarlane et al. 2011; Jones et al. 2015). Larger DNA structures can also act as such linkers by connecting several nanoparticles to larger superlattices, but they can also be used as templates onto which nanoparticles can be precisely positioned (Chao et al. 2015). Utilizing the molecular recognition properties of the DNA, DNA-based structures can serve as scaffolds for the assembly of nanoparticles using two different strategies (Samanta et al. 2015). In the first strategy, the nanoparticles are functionalized with one or more single-stranded DNA (ssDNA) oligonucleotides, which allows them to hybridize to complementary ssDNA sequences on specific positions on a pre-assembled DNA template. In the other strategy, each nanoparticle is first conjugated to a ssDNA sequence. These DNA-nanoparticle conjugates are then used to construct the tiles making up the lattice, and thus, nanoparticles are incorporated into the larger lattice structure during its assembly process.

## One-dimensional assemblies and chiral shapes

Li et al. (2004) prepared linear arrays of DNA-assisted assembly of gold nanoparticles (AuNPs). They employed DNA triple crossover molecules (TX) as templates to incorporate two biotin groups per 10-base pair (bp) hairpin loop. Next, two types of linear templates (single-layer and double-layer) were complexed with streptavidin-conjugated AuNPs to obtain linear TX arrays where the measured distance of each AuNPs were ca. 17 nm. One example of such a linear array was demonstrated by Tapio et al. (2016), as three AuNPs were assembled to form a single electron transistor with the help of three TX tiles (similarly as demonstrated by Linko et al. 2011), each containing one AuNP (linked via DNA hybridization). Beyer et al. (2005) used a different approach by employing rolling circle amplification (RCA) method to produce long-templating ssDNA strands that were hybridized with shorter biotinylated strands. Incubation with 5-nm AuNPs coated with streptavidin led into a periodic one-dimensional (1D) double-stranded DNA (dsDNA) array through biotin-streptavidin binding.

**Fig. 1:**
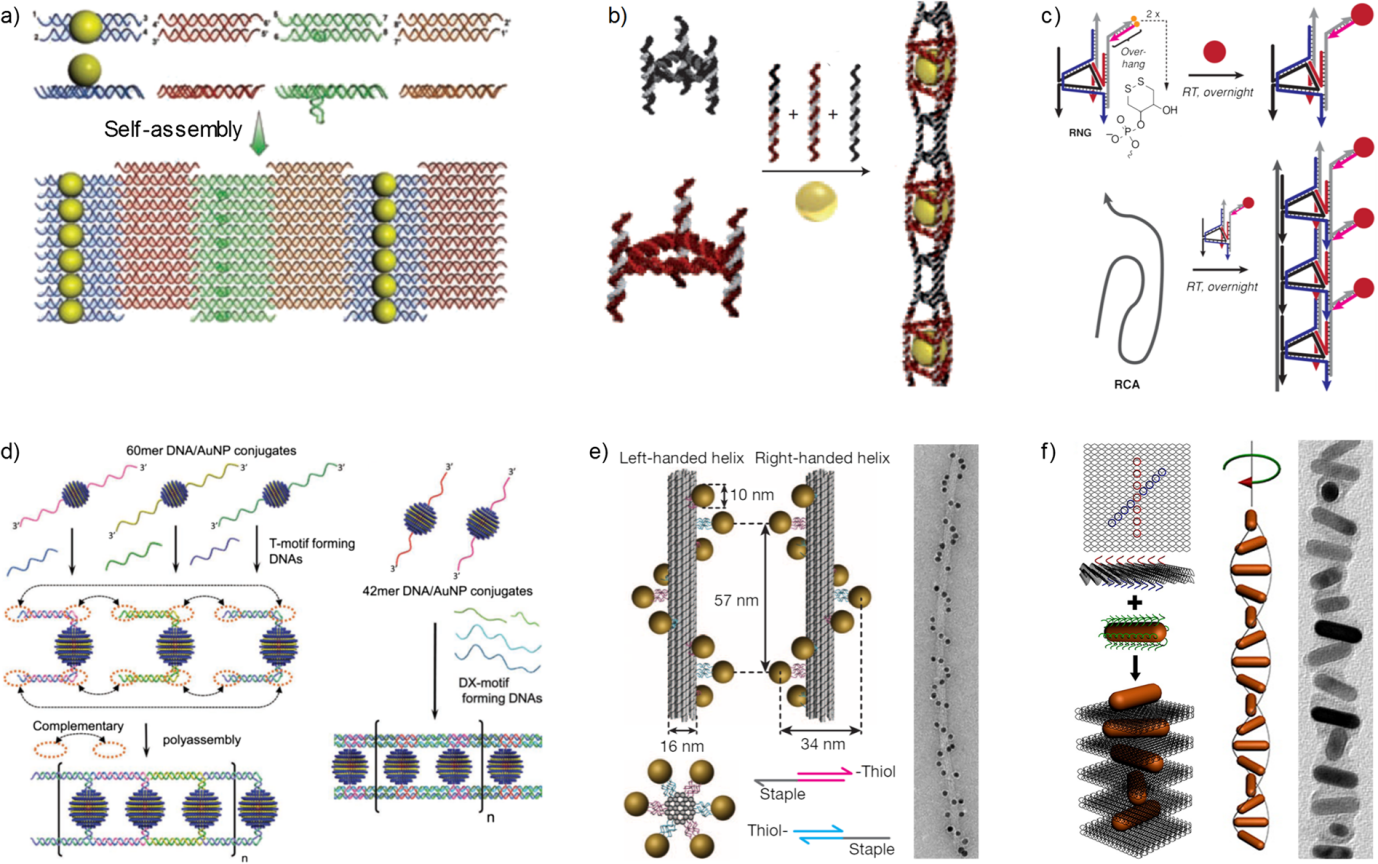
One-dimensional arrays of nanoparticles and extension to more complex configurations. **a** Double crossover (DX) tile-based assembly of gold nanoparticles (AuNPs). A 2D sheet can be further assembled into a tubular shape [reprinted with permission from Sharma et al. 2009. Copyright (2009). American Association for the Advancement of Science]. **b** Size-selective encapsulation of AuNPs using triangulated DNA tubes [reprinted with permission from Lo et al. 2010a. Copyright (2010). Nature Publishing Group]. **c** AuNP chains arranged using repeating triangular rung units [reprinted with permission from Lau et al. 2014. Copyright (2014). John Wiley and Sons]. **d** Linear and rigid AuNP (10 nm) chains formed using T- and DX-motifs [reprinted with permission from Ohya et al. 2012. Copyright (2012). John Wiley and Sons]. **e** AuNPs (10 nm) organized in left- and right-handed helical conformation for chiral plasmonics with the help of rod-like DNA origami [reprinted with permission from Kuzyk et al. 2012. Copyright (2012). Nature Publishing Group]. **f** Gold nanorods (AuNRs) assembled into left- and right-handed helical superstructures using 2D rectangular DNA origami templates [reprinted with permission from Lan et al. 2015. Copyright (2015). American Chemical Society]

Sharma et al. (2009) developed a concept to control self-assembly of DNA tubules via integration of AuNPs into double crossover (DX) DNA tiles. A two-dimensional (2D) array system was assembled from four DX tiles through sticky end interactions in a way that selected tiles displayed parallel lines of 5-nm AuNPs on top of the tile with ca. 64 nm periodicity (Fig. 1a). Close proximity of AuNPs induced strong steric and electrostatic repulsion that forced the 2D sheets to bend and rearrange into 1D tubular structures that displayed patterns of AuNPs in single or double spirals, nested spiral tubes, or stacked rings. Larger 2D arrays with 10- and 15-nm AuNPs were found to assemble predominantly into stacked ring conformations. Thus, the AuNPs act as active and structure-directing features in the formation of the tubules.

Lo et al. (2010a) fabricated triangular DNA nanotubes with longitudinal variation and alternating small (7 nm) and large (14 nm) capsules (rungs) along the structure. Size-selective encapsulation of citrate-coated AuNPs was demonstrated via simultaneous annealing of all components with double stranded linking strands. As the result, “nano-peapod” arrays, where 15-nm AuNPs were positioned at 65 nm periodicity were formed. No encapsulation was observed with 10-nm AuNPs, or by addition of 15-nm AuNPs to the pre-formed DNA nanotube, or when only small rungs (7 nm) were employed. Release of AuNPs took place when linking strands were removed by complementary “eraser strands” (Fig. 1b). Improved and more precise control over the DNA nanotube length was achieved using a finite linear DNA template, which limited the 1D growth of DNA nanotubes (Lo et al. 2010b).

Sleiman’s research group extended the above-mentioned work by designing a one-dimensional DNA nanotube (Lau et al. 2014), which was comprised of repeating triangular rung units each having five component strands, three self-complementary sticky ends and a short single-stranded binding region complementary to a long-templating RCA backbone strand (Hamblin et al. 2013). This design was used as a platform to conjugate 13-nm AuNPs into a well-defined 1D assembly via modification of a rung unit by adding two cyclic dithiol binding sites into a double-stranded overhang of each rung. Moreover, the assembly into dimers, trimers, and tetramers was demonstrated by using a backbone strand with a desired number of binding sites (Fig. 1c).

Ohya et al. (2012) reported three different motifs for the formation of 1D AuNP arrays. Using the method of Stellacci and co-workers (DeVries et al. 2007), they coated 10-nm AuNPs with a binary mixture of thiol-ligands to obtain AuNP “ripples” which were further functionalized by addition of two 5′SH-DNA 20mer (DNA strand equipped with a thiol group at the 5′ end) to form a divalent conjugate. The second conjugate, having two complementary 5′SH-DNA 20mers, was prepared and mixed with the previous one in 1:1 ratio to obtain worm-like DNA-nanoparticle arrays connected by one flexible DNA duplex. Linear AuNP arrays were obtained by using a more rigid T-motif with ternary mixture of 60mer DNA-AuNPs or a double-crossover (DX) motif (42mer) with two juxtaposed Holliday junctions joined together by two double-helical domains (Fig. 1d).

The DNA origami technique can be used to construct almost any arbitrary DNA nanostructure with nanometer precision. The robust method is based on folding a long single-stranded DNA into a predefined shape by dozens of short oligonucleotides. The method allows a variety of two- and three-dimensional shapes (Rothemund 2006; Douglas et al. 2009; Benson et al. 2015; Veneziano et al. 2016; Linko and Kostiainen 2016; Tikhomirov et al. 2017; Wagenbauer et al. 2017), and as each staple is addressable, it is straightforward to functionalize the structure in a controllable way or on the other hand, to create large arrays. These nanostructures can readily be connected to each other through sticky-end associations, and nanoparticles can be attached with thiol-modified oligonucleotides. Liedl and co-workers were among the first to use DNA origami structures for precisely arranging nanoparticles into 1D arrays (Kuzyk et al. 2012). In this work, 10-nm AuNPs were assembled into left- and right-handed nanoscale helices that act as optical polarizers using DNA origami 24-helix bundles (24HB) with precisely positioned attachment sites for ssDNA-functionalized AuNPs (Fig. 1e). Additionally, longer 1D helical arrays of AuNPs were obtained by connecting the left-handed nanohelices using complementary polymerization oligonucleotides. Lan et al. (2015) on the other hand arranged gold nanorods (AuNRs) into left- and right-handed helical superstructures utilizing 2D rectangular DNA origami templates (Fig. 1f). The AuNRs were functionalized with ssDNA sequences, and complementary ssDNA overhangs were designed on both sides of the DNA origami template in an “X”-shape manner, which allowed controlled positioning of the AuNRs with an inter-rod spacing of 14 nm and an inter-rod angle of 45°.

Furthermore, DNA origami structures have been used to arrange AuNPs into linear 1D arrays. Tian et al. (2015) constructed an octahedral DNA origami structure with AuNP attachment sites on two oppositely located vertices and used this structure as a rigid linker to connect AuNPs into well-aligned 1D arrays. Chains of AuNPs were obtained using AuNPs wrapped with DNA origami bundles into flower-shaped structures (Schreiber et al. 2016). The outer ends of two bundles, separated by 180° in the nanoflower, were further functionalized with complementary ssDNA linkers, which enabled the “nanoflowers” to assemble into 1D arrays. Liu et al. (2016a) used cross-shaped DNA origami tiles to program the 1D arrangement of AuNPs. The tiles had an AuNP attachment site in the middle, and ssDNA connector strands were added to two of the four arms. If the connector strands were added to two oppositely located arms of the structure, a linear 1D array of AuNPs was obtained, whereas a zigzag 1D array was achieved when the connector strands were added to two adjacent arms.

## Two-dimensional systems: from DX tiles to planet-satellite structures

One of the first approaches to utilize DNA templates in the construction of two-dimensional (2D) assemblies of gold nanoparticles (AuNPs) was reported by Maeda et al. (2001). Although only a limited ordering of AuNPs was obtained on the DNA network template, the study still demonstrated the power of using 2D DNA scaffolds for the precise and programmed arrangement of metal NPs. Since this early work, a number of ordered 2D arrays of AuNPs have been reported, and particularly Kiehl, Seeman, Yan, and co-workers have made significant advances towards specific arrangements of AuNPs on 2D lattices.

**Fig. 2:**
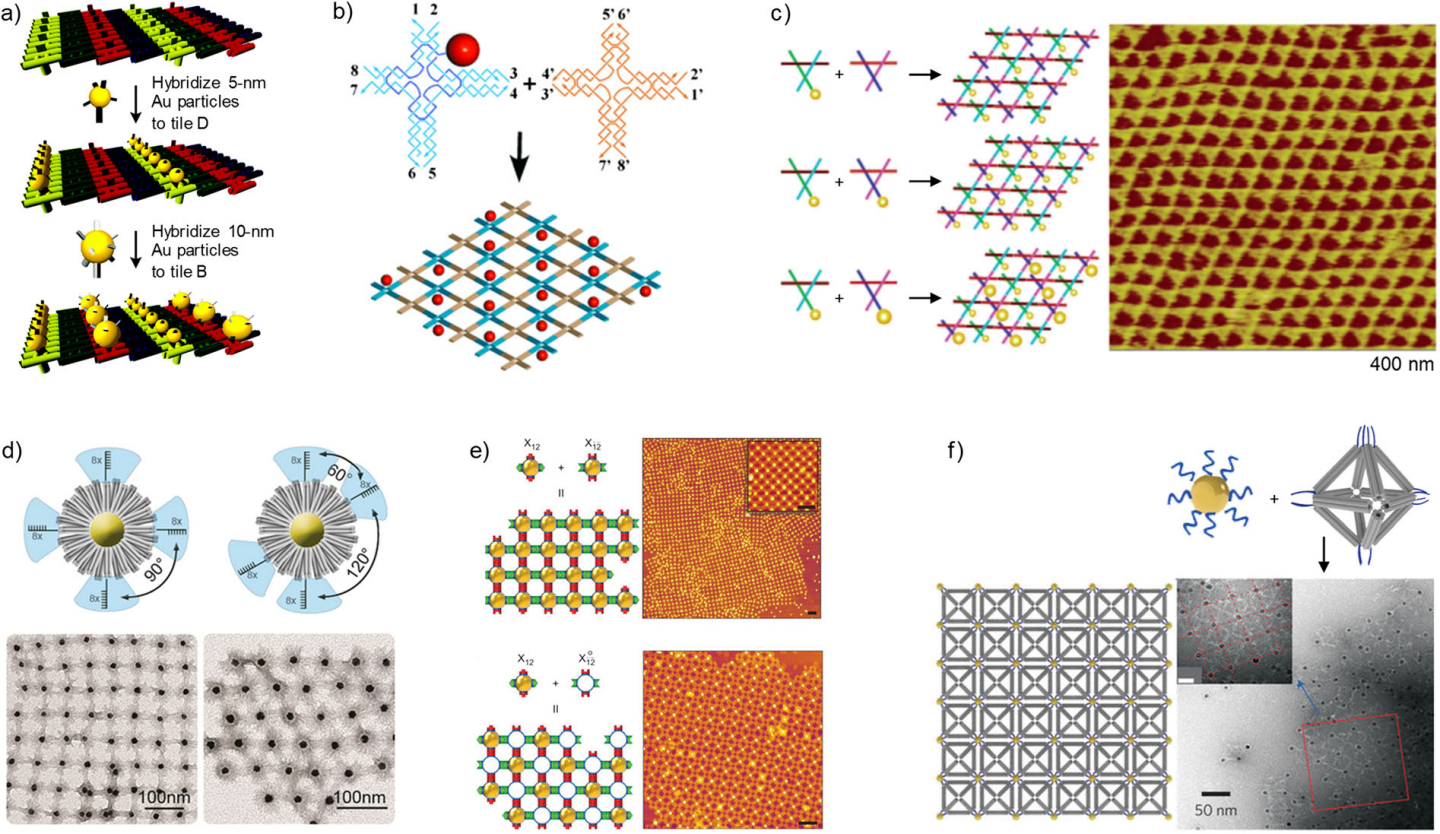
Two-dimensional nanoparticle arrays. **a** A 2D DX DNA-tile template was used to arrange 5- and 10-nm AuNPs into parallel, alternating rows [reprinted with permission from Pinto et al. 2005. Copyright (2005). American Chemical Society]. **b** Assembly of a 2D square array using AuNP-bearing DNA tiles [reprinted with permission from Sharma et al. 2006. Copyright (2006). John Wiley and Sons]. **c** AuNPs were incorporated into “tensegrity triangles” used in the assembly of 2D arrays [reprinted with permission from Zheng et al. 2006. Copyright (2006). American Chemical Society]. **d** AuNPs wrapped with DNA origami bundles formed different lattices depending on the position of the single-stranded DNA (ssDNA) linkers [reprinted with permission from Schreiber et al. 2016. Copyright (2016). American Chemical Society]. **e** 2D square lattices assembled using cross-shaped DNA origami tiles with AuNP attachment sites in the middle [reprinted with permission from Liu et al. 2016a. Copyright (2016). Nature Publishing Group]. **f** Octahedral DNA origami structures connected AuNPs into 2D square arrays [reprinted with permission from Tian et al. 2015. Copyright (2015). Nature Publishing Group]

In an initial study by Kiehl and colleagues, small AuNPs (1.4 nm) were organized into well-aligned 2D arrays on a DNA scaffold, originally reported by Liu et al. (1999) and constructed from four different double-crossover (DX) tiles with complementary ssDNA overhangs (sticky ends) (Xiao et al. 2002). Prior to the growth of the DNA crystal, the AuNPs were covalently linked to the DNA within one of the tiles, which enabled a controlled positioning of the AuNPs with inter-particle spacings of 4 and 64 nm. The same research group also reported a method for controllable alignment of AuNPs on a pre-assembled 2D DNA template attached to a mica surface (Le et al. 2004). Similarly to the previous study, the template was constructed from four different DX tiles using a design closely to one described by Liu et al. (1999), but one of the tiles contained an extended ssDNA overhang (dA_15_) instead of a covalently linked AuNP. Further, 6-nm AuNPs were functionalized with multiple strands of 3′-thiolated DNA (dT_15_) designed to hybridize to the complementary ssDNA overhangs on the DNA template, which allowed precise arrangement of AuNPs in large micrometer-sized rows with a spacing of 63 nm between adjacent rows. Kiehl and co-workers later extended this work, showing that the same principle can be used to precisely arrange 5- and 10-nm AuNPs into parallel, alternating rows with a spacing of 32 nm (Pinto et al. 2005) (Fig. 2a). By coating the two types of AuNPs with different 3′-thiolated DNA strands, the sequence selectivity of DNA was effectively exploited to position the AuNPs with remote cross-contamination between the AuNP rows. Later, similar self-assembled DX DNA-tile templates have been used to arrange streptavidin-coated quantum dots into regular 2D arrays (Sharma et al. 2008). Yet, another approach has been presented by Ke et al. (2014), where parallel chains of AuNPs and parallel AuNP monolayers have been assembled on DNA crystals based on the ssDNA tile strategy (Ke et al. 2012).

In addition to these works, AuNPs have been organized into periodic square lattices on 4 × 4 cross tile-based 2D DNA nanogrids (Zhang et al. 2006; Carter and LaBean 2011). Adopting the previously described strategies of AuNPs functionalized with ssDNA (dT_15_) that hybridize to complementary “target” sequences (dA_15_) on the DNA template and construction of 2D nanogrids (Yan et al. 2003; Park et al. 2005), Zhang et al. (2006) positioned 5-nm AuNPs into 2D square lattices in a controlled manner with a center-to-center inter-particle spacing of 38 nm. Carter and LaBean (2011) utilized a different strategy and arranged 5-nm AuNPs on the nanogrid in which a high-affinity gold binding peptide was covalently conjugated to one of the oligonucleotides used to construct the nanogrid tiles. This work clearly demonstrated that peptide-directed assembly is viable alternative to thiol chemistry in DNA-templated assembly of AuNPs.

Yan and colleagues demonstrated that well-defined periodic arrays of 5-nm AuNPs can be constructed also in a one-pot reaction by incorporating the AuNPs into the nanogrid lattice during its assembly (Sharma et al. 2006) (Fig. 2b). In this strategy, an AuNP-bearing ssDNA sequence is first used as a building material for the construction of a single DNA tile, which is subsequently assembled with other DNA tiles to form a lattice structure. The spacing between adjacent AuNPs can be precisely controlled by altering the dimensions of the DNA tile. A more complex, well-ordered pattern of AuNPs was achieved by Seeman and co-workers by conjugating AuNPs to ssDNA used in the assembly of two different three-dimensional double crossover (3D DX) triangle motifs (Zheng et al. 2006) (Fig. 2c). Three different periodic 2D arrays of 5-nm AuNPs and 5- and 10-nm AuNPs were obtained by connecting the triangle motifs by specific sticky end associations using the approach by Liu et al. (2004).

Schreiber et al. (2016) demonstrated the feasibility of using DNA origami structures in fabrication of 2D lattices with different symmetries using the already mentioned “nanoflowers” in which the AuNPs are wrapped with DNA origami bundles into flower-shaped structures (Fig. 2d). ssDNA linkers were selectively added to the outer ends of chosen bundles of these “nanoflowers,” which allowed these nanoflowers to assemble into different 2D lattices depending on the number of attachment sites and their position. Likewise, Liu et al. (2016a) used the mentioned cross-shaped DNA origami tiles with AuNP capturing strands in the middle to program the 2D arrangement of AuNPs (Fig. 2e). A large variety of well-defined planar architectures and arrays were fabricated using a lock-and-key mechanism and with different combinations of these cross-shaped DNA origami tiles having ssDNA connector sequences at one, two, three, or all of the four arms. AuNPs have also been controllably arranged onto 2D honeycomb lattices that have been assembled from DNA origami hexagon tiles (Wang et al. 2016).

A different method for constructing higher-ordered lattice arrangement of nanoparticles is to use DNA origami nanostructures as rigid linking elements that connect the nanoparticles with each other. Schreiber et al. (2014) used DNA origami nanotubes with attachment sites at the ends to assemble metal nanoparticles and quantum dots into hierarchical nanoclusters with a “planet-satellite”-type structure. They further demonstrated that the nanoclusters formed closed-packed lattices upon slow drying on solid faces, indicating that the nanoclusters were uniform in size. Tian et al. (2015) later constructed an octahedral DNA origami frame with AuNP attachment sites on the vertices, which allowed enhanced control over positioning the AuNPs (Fig. 2f). Utilizing this octahedral DNA origami structure as linking element, ordered 2D arrays were assembled by attaching AuNPs only to specific vertices of the octahedra as connecting sites.

## Three-dimensional lattices: towards novel materials

**Fig. 3:**
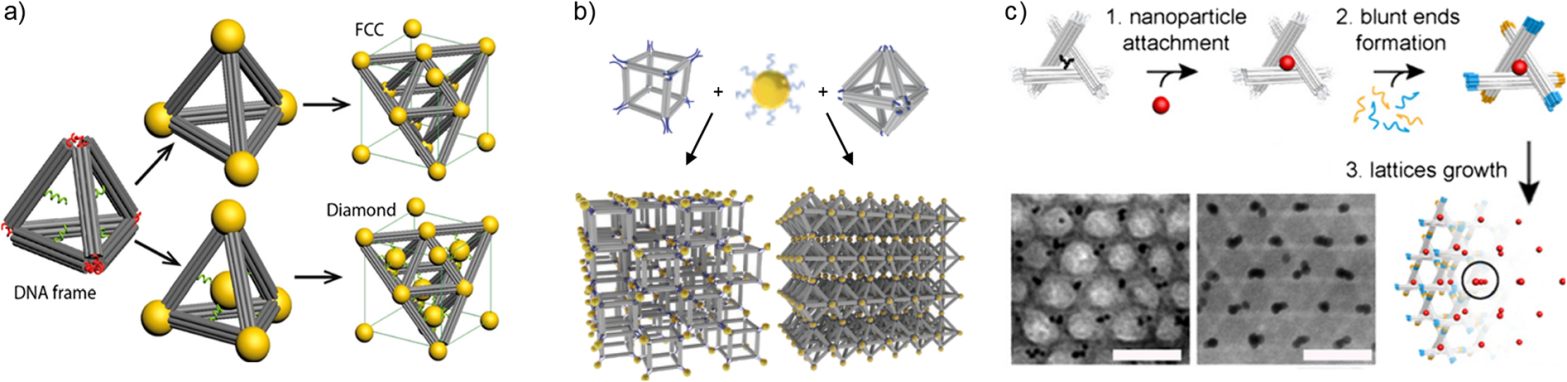
Three-dimensional AuNP lattices assembled using DNA origami frames. **a** Face-centered cubic (FCC) and diamond lattices obtained from a tetrahedron-shaped DNA origami frame (Liu et al. 2016b) [adapted and reprinted with permission from Hong et al. 2017. Copyright (2017). American Chemical Society]. **b** Various superlattices constructed using different polyhedron DNA origami frames [reprinted with permission from Tian et al. 2016. Copyright (2016). Nature Publishing Group]. **c** Rhombohedral lattice obtained from DNA origami-based “tensegrity triangles” [reprinted with permission from Zhang et al. 2017]

The self-assembly of nanoparticles into predefined three-dimensional (3D) lattices is a formidable challenge. However, the use of DNA origami frames has emerged as a promising solution. DNA origami frames are rigid and have well-defined geometries. ssDNA strands extending from the structures provide essential connecting points for nanoparticles needed for the crystal growth. The research group of Gang constructed different 3D AuNP superlattices using a tetrahedron-shaped DNA origami frame with AuNP attachment sites at the vertices and inside the tetrahedron (Liu et al. 2016b) (Fig. 3a). If the AuNPs, with a core diameter of 14.5 nm was attached only to the connecting sites at the four vertices, an ordered open face-centered cubic (FCC) lattice was obtained whereas AuNPs placed inside the tetrahedron results in a cubic diamond lattice formation. Further, an interchange to smaller AuNPs (core diameter of 8.7 nm) at the connection sites or both at the connection sites and inside the structure resulted in a zinc blende respective “wandering” zinc blende type of lattice. In a subsequent study, the same group constructed a variety of 3D AuNP superlattices using different polyhedron DNA origami frames with AuNP connecting sites at their vertices (Tian et al. 2016) (Fig. 3b). A FCC lattice was obtained when an octahedral DNA origami frame and ssDNA-coated AuNPs (core diameter of 10 nm) were employed. Accordingly, a simple cubic lattice was obtained from a cubic DNA origami frame, a body-centered-tetragonal (BCT) lattice from an elongated square bipyramidic DNA origami frame, and a simple hexagonal (SH) lattice from a prism-shaped DNA origami frame. Moreover, Zhang et al. (2017) demonstrated that a crystalline 3D rhombohedral lattice can be assembled from DNA origami-based “tensegrity triangles” in addition to a 3D rhombohedral AuNP lattice obtained by a site-specific positioning of AuNPs on the tensegrity triangles before lattice growth (Fig. 3c).

Recently, Liu et al. (2017) reported a construction of ordered 3D lattices of AuNPs without rigid DNA origami frameworks. In this study, short 6-helix bundle (6HB) DNA structures (21 nm in length) with ssDNA attachment sites at both ends were used to assemble ssDNA-coated AuNPs into different configurations depending on the effective size of the AuNPs. For a small effective AuNP sizes and low stoichiometric ratios of 6HB DNA rods to AuNPs, only disordered arrangements were formed. However, an unexpected transition from disorder to hexagonal close-packed (HCP) and further to face-centered cubic (FCC) lattices occurred when the effective AuNP size was gradually increased, and the 6HB rod to AuNP ratio was at least 5:1.

## Conclusions and future perspectives

Majority of the reported literature concerning bottom-up or top-down fabrication using DNA templated assembly of metal nanoparticle superlattices deal with AuNPs for a simple reason, superior stability of DNA-AuNP conjugates and ease of modifications compared to, e.g., silver or other metal nanoparticles which have a tendency to oxidize and form irreversible aggregates in high salt concentrations (Liu et al. 2014).

Nevertheless, silver nanoparticles (AgNPs) have other advantages compared to AuNPs: they have different localized surface plasmon resonance (LSPR) frequencies because of their higher inter-band transition threshold energy (for Ag 3.9 eV, for Au 2.4 eV). For AuNPs, considerable absorptive losses at < 500-nm wavelengths are observed, and these cause damping of the LSPR. In comparison, AgNPs have sharp plasmon resonances with larger extinction cross-sections, and these resonances can be extremely useful especially in molecular detection applications. AgNPs have successfully been conjugated with chimeric phosphorothiolated DNA (ps-po DNA) and precisely positioned onto DNA origami templates (Pal et al. 2010; Eskelinen et al. 2014). Further, Zhang et al. (2013) have demonstrated that a large diversity of hydrophobic-ligand-capped nanoparticles can be functionalized with DNA in a two-step reaction: first, the nanoparticles are coated with an amphiphilic polymer and later functionalized with DNA strands using strain-promoted azide-alkyne cycloaddition. In a proof-of-concept study, they coated oleylamine-protected CdSe/ZnS core-shell quantum dots, dodecanethiol-functionalized AuNPs, oleic-acid-protected iron oxide nanoparticles, and oleylamine-protected platinum nanoparticles with ssDNA using this strategy and assembled the newly coated nanoparticles into face-centered cubic (FCC) and body-centered cubic (BCC) crystal structures using additional, complementary DNA linker strands. Furthermore, Calais et al. (2017) have grafted aluminum and copper oxide nanoparticles using streptavidin and biotinylated ssDNA to enhance dispersion and stabilization in aqueous media. Chen et al. (2017) have prepared platinum supraparticles (PtSPs) having a valence-controlled core-satellite structure assembled together via complementary ssDNA strands, which were subsequently deposited on a rectangular DNA origami to form a finite linear array of PtSPs.

In addition to abovementioned techniques, DNA shapes can also be used for creating designer nanoparticles. Helmi et al. (2014) and Sun et al. (2014) have shown how to use DNA origami shapes as molds for guiding metal nanoparticle growth in solution phase. With this technique, one can synthesize almost arbitrarily shaped metal nanoparticles. For example, Sun et al. demonstrated customized gold and silver cuboids (sub-25-nm dimensions) with various cross-sectional shapes. Interestingly, programable interfaces of the molds enable formation of larger assemblies of these customized metal nanoparticles. For example, Bayrak et al. (2018) have successfully synthesized conductive 1D nanowires by stacking multiple molds together. Moreover, Shen et al. (2015b, 2018) have shown that by combining DNA origami shapes with conventional lithography methods, one can fabricate metal nanoshapes on various substrates with 10-nm feature sizes (dimensions of the structures 10–100 nm). The method is based on forming DNA-origami-shaped openings on silicon oxide layer in a chemical vapor deposition process and subsequently using that layer as a stencil or mask in the following lithographic steps. This enables customized fabrication with different metals, such as gold, silver, and copper. Shen et al. (2018) have demonstrated the method by fabricating for example crosses and bowtie-shapes with intriguing plasmonic properties at the visible wavelength range. So far, these nanostructures are randomly oriented on a substrate but the technique has a potential to enable large well-ordered metal lattices.

In summary, DNA-based motifs and complex assemblies provide an excellent foundation for organizing nanoparticles in a user-defined and controllable way. DNA nanostructure-directed nanoparticle assemblies have so far been developing hand in hand with the evolving field of structural DNA nanotechnology. The shape space of DNA motifs has expanded from the simple designs (branched junctions, tiles, rungs etc.) to more complex geometries (for example, 2D and 3D scaffolded origami (Rothemund 2006; Douglas et al. 2009; Veneziano et al. 2016), single-stranded tile strategies (Ke et al. 2012; Ong et al. 2017), and gigadalton-scale origami (Wagenbauer et al. 2017). As the user-defined and customized DNA structures have recently become widely accessible (Nummelin et al. 2018) and rather inexpensive (Praetorius et al. 2017), this development enables formation of large-scale superlattices (Zhang et al. 2017) and other systems that could find interesting uses as optically active components, plasmonic devices, and metamaterials.
